# cuticleDB: a relational database of Arthropod cuticular proteins

**DOI:** 10.1186/1471-2105-5-138

**Published:** 2004-09-28

**Authors:** Christiana K Magkrioti, Ioannis C Spyropoulos, Vassiliki A Iconomidou, Judith H Willis, Stavros J Hamodrakas

**Affiliations:** 1 Department of Cell Biology and Biophysics, Faculty of Biology, University of Athens, Athens 157 01, Greece; 2 Department of Cellular Biology, University of Georgia, Athens, GA 30602, USA

## Abstract

**Background:**

The insect exoskeleton or cuticle is a bi-partite composite of proteins and chitin that provides protective, skeletal and structural functions. Little information is available about the molecular structure of this important complex that exhibits a helicoidal architecture. Scores of sequences of cuticular proteins have been obtained from direct protein sequencing, from cDNAs, and from genomic analyses.

Most of these cuticular protein sequences contain motifs found only in arthropod proteins.

**Description:**

cuticleDB is a relational database containing all structural proteins of Arthropod cuticle identified to date. Many come from direct sequencing of proteins isolated from cuticle and from sequences from cDNAs that share common features with these authentic cuticular proteins. It also includes proteins from the *Drosophila melanogaster *and the *Anopheles gambiae *genomes, that have been predicted to be cuticular proteins, based on a Pfam motif (PF00379) responsible for chitin binding in Arthropod cuticle. The total number of the database entries is 445: 370 derive from insects, 60 from Crustacea and 15 from Chelicerata. The database can be accessed from our web server at .

**Conclusions:**

CuticleDB was primarily designed to contain correct and full annotation of cuticular protein data. The database will be of help to future genome annotators. Users will be able to test hypotheses for the existence of known and also of yet unknown motifs in cuticular proteins. An analysis of motifs may contribute to understanding how proteins contribute to the physical properties of cuticle as well as to the precise nature of their interaction with chitin.

## Background

One particular family of cuticular proteins constitutes one of the largest multigene families known in insects [1]. Unrelated cuticular proteins are also numerous within a single species [2,3]. This diversity of cuticular proteins is extraordinary when one considers that chitin, the other principal constituent of cuticle, is a simple filamentous polymer of N-acetylglucosamine. Over 60 sequences have been obtained from proteins extracted from arthropod cuticles freed from adhering cells, primarily through the work of Svend Andersen and his colleagues in Copenhagen. An additional 9 have been extracted from cuticle and had their N-terminal sequences determined [2,3]. These verified cuticular protein sequences revealed motifs, unique to arthropod proteins, that have made it possible to classify sequences that came from cDNAs and genomes as cuticular proteins. In addition to sequence determination, studies of cuticular proteins have emphasized spatial distribution and expression in different developmental stages (reviewed in [2,3]). Consequently, a wealth of information exists. We have used and organized this information in a relational database, named cuticleDB, the first database of arthropod cuticular proteins. The current total number of entries is 445, including proteins from 6 orders of Insects, 2 orders of Crustacea, and 2 orders of Chelicerata. This first version of cuticleDB is restricted to structural proteins of the cuticle; enzymes active in sclerotizing (tanning) or digesting cuticle and proteins involved in defense and pigmentation have been omitted.

The database nomenclature is based either on the names given by those who deposited the sequences or on codes assigned by genome projects. Thus, we have retained the existing names/codes for the convenience of the users.

## Construction and content

### Data collection

The data collection has been basically done in two ways. First, by submitting appropriate keywords (*cuticle*, *exoskeletal*, *carapace*) to the Protein databases of Entrez and Uniprot (release 1.8) [4] we collected a number of entries, which were manually filtered. Results from the two databases were checked to eliminate duplicates. Secondly, we obtained genome data for *Anopheles gambiae *and *Drosophila melanogaster*, from Ensembl [5] and EBI, respectively. These are currently the only Arthropods with annotated genomes. We searched these genomes for a Pfam motif, PF00379, setting as cutoff the recommended gathering cutoff of the corresponding Pfam entry [6]. This motif has been shown to be responsible for chitin binding [7] and most probably adopts a precise, well-defined structure [8,9]. A short version of this motif was first recognized by Rebers and Riddiford in 7 cuticular proteins [10], and, as more sequences became available [11], was widely recognized. The initial consensus was 35 amino acids long, but now encompasses 68 residues as sequence similarity was recognized at its amino-terminus and the carboxy-teminus was shortened. This 68 amino acid region, named the "extended R&R consensus" is what is recognized by PF00379 ([2,3]and references therein).

In order to ensure that our data collection is complete, we scanned all protein sequences of Uniprot (release 1.9) for PF00379. Again manual filtration was required. In addition to PF00379, other motifs have been described in cuticular proteins, some are found along with PF00379 while others define other families of cuticular proteins [2,3]. All recognized cuticular protein motifs were used to construct the database.

The data for our database was obtained by parsing the fields Definition, Accession, GI from Version, Organism and Origin from the Entrez entries. From the Uniprot entries we used Primary accession number, Protein name, Origin of the protein, Cross-references and Sequence information. This retrieval was done with Perl scripts. Additional information, concerning temporal and local expression of the proteins or corresponding mRNAs, was drawn from literature.

### Implementation

The data have been organised based on a relational model and is stored in a PostgreSQL database system. The user has supervisory access through our Apache web-server. The database is managed by an interferential software, written in Java, which tends to settle any web-server's query. Also, it implements a homemade computational tool that performs motif search as described below.

### Data retrieval

**Figure 1:**
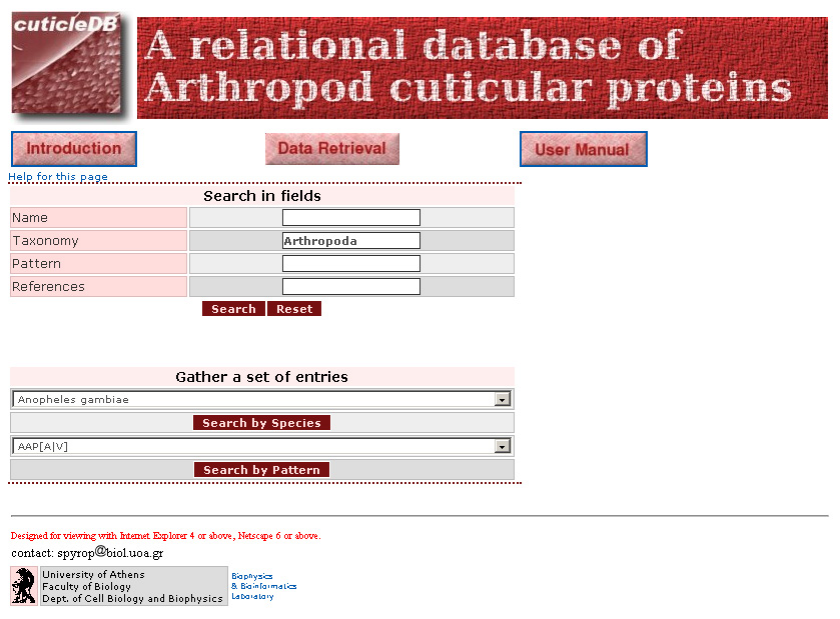
**The Data Retrieval page of cuticleDB. **The query can be done either by entering a word in the search fields Name, Taxonomy, Pattern and References or by gathering a set of entries that share a motif or derive from the same species. In this figure a query was made for all entries containing the word 'Arthropoda' in their Taxonomy field. This happens to be one of the appropriate queries for getting all cuticleDB entries.

The main page of cuticleDB includes the following interfaces: Introduction, Data Retrieval, User manual and Contact. On clicking the Data Retrieval icon, users are presented with the search interface of the database. The query can be done in two ways: either by searching in fields or by gathering a set of proteins (Figure 1).

The separate fields in which the user may search are Protein name, Taxonomy, references in other databases (the user may submit Entrez GenInfo Identifier, Entrez Accession Number, Uniprot AC, Flybase ID, Ensembl code, Interpro AC or Pfam AC as a query) and the protein sequence. The protein sequence can be searched against any pattern according to the user's imagination and, therefore, hypotheses for novel motifs can be tested. This is performed by a separate, homemade tool that has been integrated in cuticleDB and which gives the user the opportunity to detect new motifs in cuticular proteins. The integration of this tool is of importance especially in a database such as this, given the significance of motifs not only in cuticular proteins, but in structural proteins in general.

Users can gather all protein entries from a single species (35 species are included in cuticleDB) or all protein entries whose protein sequence contains one of a series of motifs. However, this series of motifs has been pre-selected by the constructors of the database and cannot be modified by the user. The selection criterion was the frequency of appearance of these motifs in the literature. The most commonly found motifs were searched against all protein sequences of the database and have properly been assigned to each entry.

### Description of an entry

**Figure 2:**
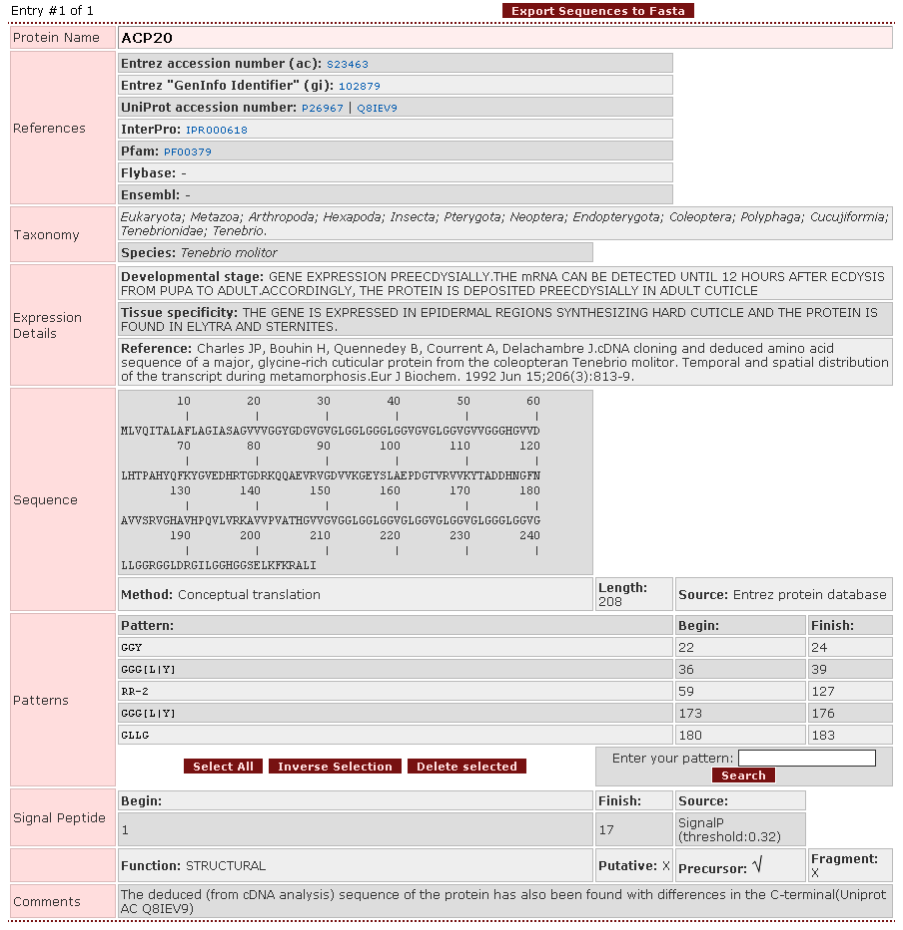
**A detailed view of a cuticleDB entry. **This contains a number of fields: Protein Name, References to other databases, Taxonomy, Expression Details, Protein Sequence and its Length, Database-Source of the sequence and the method by which the sequence was obtained. The field Patterns shows all motifs found in the protein sequence, together with their start and end positions. Users can search for their own motifs, as well. The fields Signal peptide, Fragment, Putative and Comments follow. The entry of the figure corresponds to protein ACP20 from *Tenebrio molitor*. It was selected from the Result set, that appeared after the query with the word 'Arthropoda'.

A typical cuticleDB entry contains the following fields: Protein Name, References to other databases (Entrez Protein Database, Uniprot, Interpro, Pfam, Flybase, Ensembl), Taxonomy, Expression Details, Protein Sequence and its Length, Database-Source of the sequence and the method by which the sequence was obtained (Figure 2). The field 'Expression Details' supplies the user with information about the anatomic region where each protein has been detected or the tissue where the corresponding mRNA is expressed, as well as the developmental stage in which the protein/mRNA appears. This field is usually accompanied by literature-citations. Moreover, another field named Patterns shows all patterns that have been searched for and found in the protein sequence, together with the start and end position of each. A text-box where the user can write his/her pattern is also available. If the user pattern matches the sequence, it is appended to the list of the predefined patterns. It remains there, as long as the user's session lasts. Also present are a field giving the known or predicted signal peptide and fields indicating whether the protein is putative, preliminary or fragment.

### Taxonomic distribution of the entries

Taxonomic data are taken from Entrez. The total number of entries in cuticleDB is 445. These proteins are distributed in the three large taxa: Insecta (370 entries), Crustacea (60 entries) and Chelicerata (15 entries). The database includes entries from 6 orders of the class Insecta: Diptera (258 entries), Lepidoptera (39 entries), Orthoptera (37 entries), Hemiptera (6 entries), Coleoptera (22 entries), Dictyoptera (8 entries). The large number of proteins in Diptera is due to the inclusion of cuticular proteins from the two genomes (*D. melanogaster*, *A. gambiae*). The only verified cuticular proteins are those where the complete protein sequence or a unique N-terminal region was determined from a protein extracted from a cleaned cuticle or where a specific antibody reacted with proteins in cuticle or extracted from it. Finding mRNA in the epidermis is presumptive evidence that a protein is cuticular. The majority of cuticular proteins in this database were designated as cuticular proteins based on their sequence similarity to authentic cuticular proteins. Such proteins where sequence is the sole criterion for assignment are marked as "putative" in the database. Furthermore, at present, the annotation of the proteins of *A. gambiae *is preliminary. Many proteins are missing signal peptides, other clearly have been incorrectly assembled. Such sequences are marked as preliminary as well as putative. This database will be continuously updated at regular intervals to accommodate annotation.

The distribution of the proteins in the subphylum Crustacea is 59 entries from the order Decapoda, and 1 entry from the order Sessilia, whereas the distribution in the subphylum Chelicerata is 5 entries from the order Araneae and 10 from Xiphosura.

### Motif distribution

Apart from collecting and organizing data, this database also contains results of experimental computational work. Based on the classification of the "extended R&R" motif into two main types, RR1 and RR2 [12], which, at present, appears to correlate with their presence in proteins from soft and hard cuticles respectively, we built a Profile Hidden Markov Models for the two types. For this purpose we used the HMMER software package (Version 2.3.2) [13] utilizing its hmmbuild function. As an input to this function we used an alignment derived from 14 RR1 protein sequences from *D. melanogaster *for the RR1 HMM and an alignment derived from 9 RR2 protein sequences from the same species for the RR2 HMM (suitably selected from reference [3]). Both of the alignments were restricted to the area of the 'extended R&R consensus', thus they did not include the whole sequences.

Subsequently, we used these Profile Hidden Markov Models as a prediction tool for classifying the cuticular proteins into two groups RR1, RR2. The prediction was in agreement with the literature as far as the known RR1 and RR2 proteins are concerned. The total number of RR1 and RR2 proteins in cuticleDB are 132 and 148, respectively. The start and end positions of the two motif-types are shown in the corresponding entry of each protein. A smaller class, RR-3, with 75 conserved residues was also identified by Andersen [14].

We have also studied the appearance of another motif: AAP(A/V). This small, hydrophobic tetrapeptide has been found to occur mainly in proteins of hard cuticles [2,3], where the water content is low and the sclerotization is intense. We have found that the AAP(A/V) motif occurs in 43% of the RR2 proteins, whereas only in the 12% of the RR1 proteins of cuticleDB.

## Utility and discussion

The most severe problem of genome projects to date is that of correct annotation. So, accurate and specialized databases as cuticleDB with its description of highly conserved motifs will be of help to genome annotators. Therefore, cuticleDB can be used as a basis for annotating new cuticular proteins by similarity in future Arthropod genome projects.

cuticleDB can also be utilized in molecular research as well, due to its focus on motif appearance. Cuticular proteins, as is the case with all structural proteins are marked by the presence of characteristic motifs. Some motifs are repeated within a protein sequence, whereas others appear only once. cuticleDB has been designed in such a way that the user can have a complete view of motif occurrence in the sequence of each protein entry. First, each entry shows the exact position of the most common cuticle motifs in the protein sequence. Secondly, the user is given the opportunity to search the sequence for novel motifs and therefore, test hypotheses for the existence of new patterns. Subsequently, hypotheses for possible interactions between cuticle macromolecules (either proteins with chitin or proteins with proteins) can be tested. Moreover, our results of the RR1 and RR2 predictions can be used as a guide for identifying a certain protein as coming from either soft or from hard regions of the cuticle. Most importantly, the information about the RR1 and RR2 distinction can be used for studies of cuticle's mechanical properties. As RR1 and RR2 proteins appear in soft and hard cuticles respectively, which means that the former interact with chitin more loosely than the latter, one can gain an insight in cuticle's molecular construction combining our data on the sequences of RR1 and RR2 proteins with some experimental work. Moreover, one could use the Expression Details, namely where and when each protein is expressed, when studying the differential construction of the cuticle among different developmental stages or among different regions of a single cuticle.

## Conclusions

The goal of cuticleDB constructors was the collection of all cuticular protein sequences that have appeared to date and their detailed and correct annotation. The better the organisation of the data, the easier the work will be for researchers dealing with cuticle and structural proteins in general. cuticleDB will help them to answer questions like : 'What kind of proteins appear in hard cuticles?' 'Why do RR2 proteins interact with chitin more tightly than RR1 proteins?' 'Which motifs contribute to protein-protein interaction in the cuticle?' 'From which stage can a certain protein be extracted?' Furthermore, it is hoped that, detection of common properties of these proteins, as well as recognition of important differences that are responsible for cuticle's complexity and important functions will be facilitated by its existence.

Last but not least, it is hoped that this database will be of help to genome annotators in the near future as more arthropod genomes become available.

## Availability and requirements

cuticleDB was created and is maintained in the Department of Cell Biology and Biophysics, Faculty of Biology of the National and Kapodistrian University of Athens. It is freely available at the URL: . An e.mail biodb@biol.uoa.gr may also be used for comments, corrections and further data (sequence) submission.

## List of abbreviations

RR1: The extended Rebers and Riddiford Consensus, type I

RR2: The extended Rebers and Riddiford Consensus, type II

HMM: Hidden Markov Model

## Authors' contributions

CKM performed the data collection, and test procedures, and also participated in the design and the implementation of the database

ICS carried out the design of the algorithms and the database,. implemented all the algorithms, and also created the web interface

VAI supervised the data collection and the tests

JHW compiled the first draft of known cuticular proteins, provided a critique of the data base during its construction

SJH coordinated and supervised the whole project, suggesting the general directions and innovative features of the database

All authors have read and accepted the final manuscript.
